# Navigating Hostile Workplaces and Educational Spaces Within Health Services and Policy Research

**DOI:** 10.1089/heq.2024.0121

**Published:** 2024-12-16

**Authors:** Taylor B. Rogers, Kevin Q. Graham, Carmen R. Mitchell, Tongtan Chantarat, Michelle J. Ko

**Affiliations:** ^1^ Department of Health Policy and Management, UCLA Fielding School of Public Health, Los Angeles, California, USA.; ^2^ Center for the Study of Racism, Social Justice, and Health, UCLA Fielding School of Public Health, Los Angeles, California, USA.; ^3^ Department of Computer Science, UCLA Samueli School of Engineering, Los Angeles, California, USA.; ^4^ Department of Health Management and Systems Science, University of Louisville School of Public Health and Information Systems, Louisville, Kentucky, USA.; ^5^ Institute for Social Research and Data Innovation, University of Minnesota, Minneapolis, Minnesota, USA.; ^6^ Division of Health Policy and Management, Department of Public Health, University of California, Davis, California, USA.

**Keywords:** workforce, structural racism, diversity, equity, and inclusion, discrimination, workplace culture

## Abstract

**Introduction:**

The representation of ethnoracial minoritized individuals in health services and policy research (HSPR) has increased in recent years. However, previous literature has exposed a need to acknowledge and attend to inequities within the HSPR workforce.

**Methods:**

To describe educational and workplace experiences that characterize diversity, equity, and inclusion (DEI) within the HSPR profession. In this qualitative study, six focus groups were conducted virtually via Zoom with 27 individuals who reported working or pursuing higher education in HSPR from December 2020 to January 2021. We sought HSPRers perspectives on DEI initiatives, work and educational environments, experiences, and climate, and recommendations for improving DEI in HSPR. We developed a structured codebook and applied a deductive approach to conduct thematic analysis.

**Results:**

Of the 27 participants, nearly half of participants identified as Black/African American (44%); most were women (81%). Three major themes emerged: (1) HSPR work and education spaces subject minoritized HSPRs to a range of exclusionary and harmful practices; (2) DEI initiatives fail to address the need for institutional change; and (3) by working with and for policymakers, HSPRs are uniquely subjected to shifting political contexts that reinforce racism.

**Discussion:**

Despite an increasing commitment to increasing the diversity of the HSPR workforce and improving equity and inclusion in the HSPR workplace, the findings suggest that more intentional and action-oriented work is needed, especially work that emphasizes inclusion and equity across various levels of the workplace.

**Health Equity Implications:**

The findings offer critical insight on necessary workplace and educational reform to develop the workforce necessary to advance population health equity and equity-oriented policy making.

## Introduction

Advancing health equity requires acknowledging and attending to inequities within the health policy and services research (HSPR) workforce. Although HSPR racial and ethnic diversity has been increasing, the profession still has considerable unfinished work: in 2021, Black, Hispanic, and Indigenous HSPRers accounted for 5.3%, 3.1%, and 0.3%, respectively,^1^ which is not representative of the U.S. population. Similar to medicine,^2,3^ the lack of diversity in HSPR impacts the representation of research questions and topics, expertise and perspectives in decision-making, and evidence available to support policymaking that reflects a variety of lived experiences and/or community needs. The field has recognized the need to recruit and retain individuals from ethnoracial groups historically and structurally excluded and cultivate mentorship to ensure diverse representation in leadership. In 2020, we observed a nationwide acknowledgment of structural racism across the U.S. and increased efforts to shift DEI-related issues in workplaces and educational spaces following the murder of George Floyd, Jr.^4^

Survey studies from other disciplines related to HSPR have further documented the role of professional and workplace climate in fostering inequitable, noninclusive environments. Ethnoracially minoritized professionals, from epidemiology to economics to medicine, have reported pervasive professional social exclusion and isolation, relative to those racialized as White.^5,6^ In a study specific to HSPR, Chantarat et al.^7^ found that members of the workforce experienced noninclusive, inequitable work environments. Over 70% of Black/African American and over 50% of Hispanic/Latinx and South Asian participants personally experienced professional discrimination. Higher proportions of structurally excluded groups perceived HSPR diversity, equity, and inclusion (DEI) efforts to be superficial and tokenistic, with an emphasis on discussion rather than implementation.^7^

This study is guided by the theory of racialized organizations, as we aim to critically analyze experiences of structurally excluded individuals within HSPR organizations, specifically the workplace and educational spaces. The theory of racialized organizations posits that formal and informal organizational processes unfairly advantage some racial–ethnic groups at the expense of others, as organizations are “constituting and constituted by racial processes” that may shape the institutional/macro level, the organizational level, and the individual/micro level.^8^ Further, racialized organizations: (1) shape agency; (2) legitimize the unequal distribution of resources; (3) treat whiteness as a credential; and (4) decouple certain formal processes in a racialized way.^8^

The current survey-based literature has set a foundational understanding of persistent exclusionary HSPR workplace and educational settings. What is not known is how these experiences emerge, their specific forms, or the impacts on those subjected to hostile work environments. This study aims to close these gaps by investigating DEI within the professional climate of HSPR and elucidating the lived experience of those from ethnoracially minoritized groups.

## Methods

### Setting, Participants, and Study Design

We conducted six virtual focus groups from December 2020 through January 2021. We used focus groups to elicit information about the range of feelings and ideas that participants have regarding a particular subject and highlight differences in perspectives between groups and individuals.^9^

We identified potential participants from the respondents to the HSPR Workplace Culture survey,^7^ which was distributed through AcademyHealth media, the 2020 Annual Research Meeting, and social media campaigns across multiple related public health, social science, and biomedical professions. We then conducted an initial targeted recruitment of survey respondents who had (1) indicated interest in participating in a follow-up focus group study and (2) identified as an ethnoracially minoritized person. We consider “ethnoracial minoritized individuals” as individuals structurally marginalized by the majority (non-Hispanic White) due to their race or ethnicity. Due to limited initial enrollment, we expanded our inclusion criteria to any survey respondent who was interested in participating in the focus group. To prioritize structurally excluded ethnoracially minoritized groups, we sent them invitations first and then recruited for the remaining spots from the broader group of interested HSPR respondents. We use the term “structurally excluded” to highlight the way that societal structures prevent some individuals from having access to their rights and ability to participate in their communities and decision-making process meaningfully. The focus groups were conducted virtually on Zoom, from 1 to 2 hours, recorded, and transcribed using Rev.com.

### Focus Group Protocol

The focus group questions centered around two domains: (1) general work environment and (2) improving DEI in HSPR. The focus group discussion guide was written by C.M. and T.R. and checked by M.K. and T.C. C.M. was the primary discussion facilitator, while T.R. took field notes and managed technological and administrative tasks. Participants received a $30 gift card.

We attempted to minimize the risk of loss of confidentiality by obtaining verbal consent, saving identifying information in a password-protected and encrypted source only accessible to the study team. Participants were assigned pseudonyms by the principal investigator to limit identification by name or gender in the analysis. Names and contact information were destroyed after the focus groups and were not used for analysis or reporting. We did not collect other identifiable information such as date of birth, state of residency, or place of employment. The protocol was approved by UCLA’s Institutional Review Board, and the findings are presented in accordance with the Standards of Reporting Qualitative Research reporting guidelines.

### Data Analysis

We conducted a codebook thematic analysis using a deductive approach, as we conducted this study with key research questions, and they guided our analytic approach. T.R. and K.G. created a structured codebook prior to analysis, using references and survey findings as source material from A.B. and C.D. The codebook consisted of 46 codes, and their definitions can be found in Table 2. The transcripts were coded by T.R. and K.G.; T.R. and K.G. recoiled the coding and created the themes. C.M., T.C., and M.K. checked and verified the thematic analysis.

### Positionality

Two members of the study team identify as Black women, one identifies as a Southeast Asian man, another identifies as an East Asian woman, and one identifies as an Afro-Caribbean man. As “outsiders within” the field of HSPR, we have integrated theoretical and methodological frameworks from our respective disciplines and with expertise from our lived experiences as trainees, researchers, and scholars focused on marginalization.^10^ This fusion of knowledge yields a “double consciousness”^11^ that affords us the ability to incorporate multiple perspectives in this study, which frames the selection of research questions, methodological approaches, and data analysis.^10^ T.R., C.M., T.C. (Ph.D.) and M.K. (M.D. and Ph.D.) are trained HSPRers.

## Results

We recruited 27 participants total across six groups. Nearly half identified as Black and/or African American, and most were women (Table 1). Two-thirds were aged 35–64 (66.6%) years old, and approximately one-third of participants were early career academics (students, postdocs, and assistant professors). In the Supplementary Data S1, we list positions and organizational types and assign pseudonyms to protect participant identities. Qualitative analysis of the focus groups identified three major themes (Tables 2–4).

**Table 1.** Health Services and Policy Research Focus Group Participants, *n* **=** 27

	*n*	%
Age		
18–34	7	25.9
35–44	9	33.3
45–64	9	33.3
65 and older	2	7.4
Racial-Ethnic Identity		
Asian	1	3.7
Black/African American	12	44.4
Hispanic/Latino	3	11.1
Middle Eastern or Northern African	1	3.7
More than one race	4	14.8
White	6	22.2
Sexual Orientation		
LGBTQI+	4	14.8
Heterosexual	22	81.5
Other	1	3.7
Gender Identity		
Men	5	18.5
Women	22	81.5
Employer Type		
Academic Institution	15	18.5
Government	2	7.4
Health Care Administration	4	14.8
Non-Academic Research Organization	3	11.1
Non-Profit	1	3.7
Did not disclose	2	7.4
Position Title (or seniority or career stage)		
Doctoral Candidate/Postdoctoral Fellow/Assistant Professor	8	29.6

*Notes:* *n* is the subsample, % are the column percentages; LGBTQI, lesbian, gay, bisexual, transgender, queer and/or questioning, intersex.

**Table 2.** Underrepresented and Minoritized Individuals Report Exclusionary and Harmful Experiences in Health Services and Policy Research Work and Educational Settings

Theme	Subthemes	Illustrative quote	Speaker
Exclusionary systemic inequities	*Inequities in allocation of time and resources*	“So, I did not even learn about any of that until my 5th and final year… how other people got into those labs. My first year, I was just told I was a full-time TA…I do not even know how they made that decision to put who were, but it just seems like the people that [had a graduate research assistant position]—they are the ones who are graduating on time, who have all the publications; they are happy and working in these labs. “	Ellis
		**“**They sent out a request (to see who wants to go to a national conference, and then they will support them to go) And you know, you’re not going to be one the first five people named…all those things, again, come down to, what’s the difference between me and these other people?”	Jesse
	*Devaluation of racism research*	**“**About 10 years ago, I tried to publish a paper where I was trying to research racism with a person’s health outcomes. And the reviewers were saying, well, you can not link those two together. It used to be the kiss of death on a paper to actually cite racism… like Health Affairs or American Journal of Public Health, or in any of the big-name journals, if you put racism in there, it was not getting published.”	Amari
		“(The reviewer) was pissed about that statement (about the existence of racism) and saying, ‘you can not make broad and sweeping comments, and you need citations.’ Did you need Jim Crow? Did you want me to cite the Civil Rights Movement?”	Sage
		“If the outcomes are going to benefit Black or brown people … there still is going to tend to be some question that will make it less likely for Black and brown people to secure the funding”	Erin
	*Devaluation of minoritized scholars*	“they gave me a (publication) number, and I had more than that number. But I still was not tenured, when the other two people were. And the year that I went up for tenure, there were no people of color on any of the three campuses at the (university) that made tenure, so they did an investigation. I do not know what happened, (laughs), but they came back and did an investigation.”	Amari
Interpersonal harms	*Abuse*	**“**it had all the patterns of what abusive relationships look like… say demeaning things and horrible things, and constantly put me down on a daily basis, but then also kind of lean on me for his emotional insecurities. And it’s as though he’s like, “Oh, I’m going to take advantage because you are a Latina,” …(after) I got out of that relationship and situation, that I realized where I was like he was really using my background against me to—to reinforce his power over me.”	Cameron
		“I was screamed at, told that I was a failure, I would never amount to anything. And I mean, he was getting physically aggressive with me. Um, and so that is when I finally came forward.”	Memphis
	*Exploitation*	“Knowing that I did grow up with undocumented immigrants, knowing that my mom’s a housekeeper and, knowing that I did not come from money or a privileged background… (my advisor, who was also department chair) knew how to exploit the fact that I can not just (fight back)… He would threaten me to kick me out of the program, he would threaten to not pay me. He would threaten me with so many things, knowing that knowing that I was vulnerable financially.”	Cameron
	*Harassment*	“I went to HR, I mean it was... CEO of the hospital, lawyers were involved, and I was retaliated against for speaking up. This was just a few months before the Me-Too movement, so I think had Me Too happened beforehand, it might have been a different experience.”	Memphis
	*Retaliation*	“My reputation was just dragged through the mud. He started lots and lots of rumors. And because he was in a position of power, like he had access to the CEO of the hospital, the chair of the department. When I was interviewed about my experiences, during this investigation, I was questioned, my mental health was questioned whether I was just, um, a weak female and was not really used to being in this male-dominated field, and that I maybe was overreacting.”	Memphis

*Notes:* These are selected quotations to illustrate theme 1: harm and exclusion within health services and policy research workplace and educational settings; TA, teaching assistant; HR, human resources; CEO, chief executive officer.

**Table 3.** Experiences of Diversity, Equity, and Inclusion Efforts (DEI) in Health Services Workplace and Education Settings

Subtheme	Illustrative quote	Speaker
Reactive to Social Movements	“I think that because of recent events, the disparities related to COVID, Black Lives Matter work. But one of the things that is really concerning is that the national class standards for culturally and linguistically appropriate care have been out since 2000 or 2016 and people still are acting like they are completely brand new. Like they have never heard of them before, and they are not putting forth any of the efforts that need to be done.”	Sage
Publicity without substantive reform or support	“Quite frankly, I do not care when you make social media posts about your DEI goals and things like that. I do not care about the fact that you appointed a woman of color or somebody of color to your board, … you are not doing anything to retain that person. The minute they step into the role, you are probably creating all types of hazards and barriers to their involvement in the process, that they end up quitting, or you end up creating a paper trail to let them go or to substantiate you wanting to let them go after X number of months or years.”	Remy
DEI workplace efforts are cyclical and impermanent	“I really been trying to figure out what in George Floyd’s death triggered a culture shift and why did not it happen with like Trayvon Martin or Tamir Rice, or even, if you really want to go back in history, why not Emmett Till up until this point?”	Paxton
New DEI efforts show promise, but implementation and sustainability remain uncertain	“We are trying to essentially create sort of, like, a match-making service where we can kind of look at folks on our end who do a certain line of work and- and meet certain kind of colleagues (of color) and try to match them with colleagues (of color) the area universities around us. It’s just started… these things kind of take a long time and some of these things may fizzle out but we have found it to be really helpful just in terms of understanding, you know, what these other researchers are doing in the area	Lucia

These are selected quotations to illustrate theme 2: diversity, equity, and inclusion initiatives fail to address the need for institutional change; COVID, coronavirus disease 2019; DEI, diversity, equity, and inclusion

**Table 4.** Political and Policy Contexts Impact Workplace Equity and Inclusion

Illustrative quote	Speaker
**“**(Some of the policies this administration) put in place have hindered our efforts. And then by trying to navigate that, it does kind of impact the relationships that we have…”	Wren
“I designed an entire module around equity, social justice, the impact of racism on health outcomes, and had the green lights from my supervisors, our funders at the Department of Health Services. But then a couple of months later, my office was dissolved, and I was reassigned… I was not actually told that face-to-face or by a phone call, I got that by text message and email. So, you can imagine how that makes me feel about standing up for social justice issues in our community.”	Drew
“We have a couple of very conservative types (on our board). We have a couple that are more on the progressive side of the spectrum. One of the challenges is, having a conversation about these issues, social determinants, race, racism those, without it immediately becoming political in a way that makes it very difficult to continue the conversation in a productive direction. Our audience is the state legislature, the executive branch and again…it is difficult to have substantive conversation and not have it become political, and that really hampers our ability to have those conversations…”	Ryan
**“**Most of my colleagues are more progressive, a few moderate colleagues who are polite and respectful to the right. But in the classroom where I teach, we have a lot of students who are on the two extremes. Makes for a very tension-filled classroom, especially right now.”	Adrian

*Notes:* These are selected quotations to illustrate theme 3: by working with and for policymakers, HSPRs are uniquely subjected to shifting political contexts that can undermine equity and inclusion

### Theme 1: Harm and Exclusion Within HSPR Workplace and Educational Settings

Participants shared adverse experiences, which can be broadly categorized into subthemes: (a) discriminatory policies and practices, both local and national; and (b) interpersonal harms, including exploitation and abuse. Participants described how the cumulative impacts of these types of experiences led to deterioration in their mental and physical health, exiting organizations, and/or considering leaving the HSPR field entirely (Table 2).

Multiple participants witnessed resources and time allocated to White peers while simultaneously being informed their requests or offers could not be met due to insufficient organizational resources; a consistent lack of transparency reinforced these practices. The experience of Jesse, a new assistant professor, offers an illustrative example. After he began his position, his department leadership informed him that they could no longer provide the start-up funding offered in his contract due to a lack of university resources:

*They are cutting down the start-up packages. But, in my contract, you promised that you’re going to give me a start-up package … They (responded), You can complain, but unfortunately, the university doesn’t have fund*s.

Within the same department, the following semester, a new faculty member, who was racialized as white, was hired with full start-up funds. Jesse shared several similar examples in which his colleagues, who were mostly racialized as White, received institutional resources to support research, teaching, and professional development, but those resources were not offered to him.

For subtheme (b), participants described repeated interpersonal harms, manifesting as abuse, exploitation, harassment, and retaliation (Table 2). Postdoctoral fellow Cameron described how their PhD advisor had leveraged fear, particularly via the exploitation of Cameron’s status as a child of undocumented immigrants with financial precarity, to ensure Cameron’s compliance and maintained a verbally and emotionally abusive relationship. Similarly, in Memphis’ prior position as an assistant professor, the health system chief executive officer subjected Memphis to sexual harassment, verbal abuse, and physical aggression. When she attempted to report these harms, both her abuser and other senior leadership threatened her professional reputation and work—from publications to grants—to instill fear of speaking out and then to enact retaliation.

When work and educational organizations actively or passively condone abuse or dismiss complaints, they inflict several harms upon individuals. Participants described how these experiences exacted severe mental and physical health costs, delayed their career advancement, and drove them to leave their organizations.

### Theme 2: DEI Initiatives Fail to Address the Need for Institutional Change

When queried about DEI initiatives, participants reported that organizational DEI efforts were frequently ineffective or actively counterproductive (Table 3). Participants characterized these efforts as “window dressing,” i.e., publicity without substantive reform or support; reactive to social movements, rather than proactive and systematic, thus contributing to the observation of this work as cyclical and impermanent.

Participants explained that they were skeptical of DEI initiatives that did little to acknowledge existing problems. They noted that efforts were often rushed and emphasized simple, quantifiable outcomes, rather than intentional processes that focused on quality and democratic decision-making. Those with more professional experience referenced past reform efforts that had also been short-lived and ineffective. A few spoke more positively of their organization’s DEI work as promising, particularly in the overdue recognition of structural racism—but noted they had not yet observed significant investment in implementation and sustainability (Table 3).

### Theme 3: By Working with and for Policymakers, HSPRs Are Uniquely Subjected to Shifting Political Contexts That Can Undermine Equity and Inclusion

Participants described several experiences that arose specifically due to the nature of their work in/with governmental agencies and policymakers. The actions of local, state, and federal governments, as well as the level of politicization of their organizations, impacted participants’ work environments (Table 4). Because many worked on health equity issues, several described how changes to a more conversative administration either limited or terminated their projects. For others, navigating the differing political interests of their stakeholders presented heightened challenges to accomplishing their goals and/or increased the level of work and educational tension. In summary, for minoritized HSPRers, shifting political contexts presented a unique challenge to maintaining equitable and inclusive environments.

### Participants’ Suggestions to Improve HSPR Workplaces and Educational Spaces

Participants offered a variety of suggestions to improve HSPR workplaces and educational settings in systemic and sustainable ways. First, participants agreed about the need to engage in critical self-reflection, reeducation on historical and contemporary systems of oppression, and a clear differentiation of concepts of DEI and racism for their HSPR peers. Second, participants recommended standardizing organizational processes to reduce unfair advantages in allocation of resources and support, and increase transparency to prevent the obfuscation of inequities. Third, participants called upon the need for processes that foster change in leadership to those who prioritize systemic DEI transformation, such as a “phase out” process to transition out longstanding leaders, who may not have the capacity, training, education, or motivation to reform workplace culture and systems. Finally, at the regional and national level, participants emphasized the need to engage and build equitable partnerships with Historically Black Colleges and Universities (HBCUs) for pathway programs, internships, and fellowships. At the institutional level, professional associations and accreditation bodies, such as the Association of Schools and Programs of Public Health, should directly engage HBCUs by waiving membership fees and aiding with program accreditation.

## Discussion

We found that health services and policy research workplace and educational settings foster processes of active exclusion, systemic inequities, and harm. They are broadly consistent with arguments that organizations and workplaces foster institutional-level racial inequities as well as individual-level experiences of discrimination and prejudice.^8^ Underrepresented professionals experience delayed or blocked promotion,^12^ redirection to administrative positions,^13^ racialized accusations of unprofessionalism,^14^ greater infringement of their professional boundaries,^14,15^ and difficulty navigating the tension between inclusion and assimilation.^14^ We draw upon two organizational theories to elaborate on how lack of equity and inclusion produce these outcomes in HSPR and thus adversely affect efforts to improve diversity: (1) the theory of racialized organizations; and (2) the characteristics of white supremacy culture.

### HSPR in Relation to the Theory of Racialized Organizations

Jesse’s experiences connect to various tenets of the theory of racialized organizations. Jesse’s agency was diminished due to the limited transparency in the university’s/department’s/institution’s decision-making, a symptom of White supremacy culture (e.g., power hoarding). Ray^8^ further explained how racialized organizations shape agency by controlling workers’ use of time. For our participants, workplaces shaped agency in a racialized fashion via the theft of time from non-White HSPRs. Time is a valuable resource and privilege; time theft is a component and result of structural racism and discrimination.^16^ In this case, time theft has long-term impacts on work and career advancement.

In Jesse’s case, he observed his organization treating whiteness as a credential, in that his White colleagues disproportionately benefitted from the organizations’ legitimizing of unequal distribution of resources. In theme 1, subtheme 2, Cameron and Memphis’ experiences were impacted by the ways their organizations legitimized unequal distribution of resources as well as diminished agency. Both tenets are salient in their subsequent experiences of retaliation after reporting abuse, exploitation, and harassment, as well as the intersectional oppression of sexism.

### HSPR in Relation to the Characteristics of White Supremacy Culture

White supremacy culture is the widespread ideology in society that teaches us overtly and covertly that whiteness holds values and whiteness is value. This ideology is reflected in the characteristics, and their definitions can be found in the online Supplementary Data S1. Many participants shared that scholarship on racism and minoritized communities was devalued, and while acceptance in this area of work is growing, scholars were continually questioned (Table 3). This phenomenon reflects characteristics of white supremacy culture, characteristics of one right way and worship of the written work. One right way is the belief that there is one right way to do things and assumes that anyone who does not adapt to “fit” is wrong. Worship of the written word is the habit of honoring what is written and only what is written to a narrow standard, even if what is written is incorrect. Our findings offer insights into the institutional mechanisms that contribute to inequities in funding and publication,^17–21^ including denialism and minimization within the peer-review process and overall organizational priorities.

Cameron and Memphis’ experiences highlight White supremacy culture’s fear of (open) conflict, which is based upon the unspoken assumption that those in formal and informal power have the right to comfort.^22^ When abusers have leadership positions within an entrenched organizational hierarchy, they can retain the power to exact retaliation at the level of institutions, beyond individual interactions (i.e., defensiveness and denial). Defensiveness and denial are used to delay/remove agency of subordinates and preserve comfort for those in power. In Cameron’s case, the department leadership denied the abuse experienced and acted defensively by suggesting that acquisition of grant funding can excuse abusive behavior. This is in alignment with previous work in the nonprofit domain of White supremacy reflected in their organizational culture and structure.^23,24^

Per theme 2, the rushed nature of DEI efforts aligns closely with the White supremacy characteristics of urgency, quantity over quality, and progress is bigger. A common thread of these characteristics is the sacrifice of minoritized individual interests to maintain or even improve conditions for their White counterparts.^22^ Not only does this sense of urgency make it difficult to engage in work in an inclusive, democratic, and intentional way, it also reinforces and enhances existing power hierarchies/imbalances, in turn, further diminishing minoritized groups.

Because HSPRers work in/with policymakers, political and policy contexts can perpetuate institutional racism, both restricting the work and fostering internal organizational racism (Theme 3). In many cases, HSPRers work in highly politically polarized environments; many participants work in places entrenched in the either/or and binary characteristics of white supremacy culture (Table 4). Similarly, participants shared experiences that resembled paternalism and perfectionism, and that fraught political context dampened appreciation for their work. These characteristics of White supremacy culture reinforce racialized organizations’ ability to diminish the agency of racial groups (e.g., the experiences of Wren, Ryan, and Drew) and legitimize the unequal distribution of resources (e.g., Drew) in HSPR workplaces and educational spaces (Table 4).

### Implications for HSPR: Moving Forward

Our findings related to political climate are particularly salient at present, as DEI initiatives are facing legal challenges, state and local governments are eliminating DEI positions, budgets,^25,26^ and the use of tools, from race considerations^27^ in admissions to diversity statements in hiring.^28^ Ethnoracially minoritized HSPRers may face increasing professional precarity, even though they remain relatively underrepresented and overall gains in DEI have been limited. Now is the time for HSPR organizations to redouble, not back down, on their DEI efforts.

### Strengths and Limitations

Our findings provide detailed elaboration on previously reported survey findings, thus also highlighting opportunities for change. We conducted focus groups during a period of heightened national attention to racial justice—HSPR experiences may have changed since then. However, collecting data during this time period also offers a context in which many in HSPR were engaged in critical assessment of racism within the profession. It would be particularly informative to compare as political and institutional conditions have evolved since. Our study is limited to considerations of ethnoracial diversity, and we acknowledge that DEI across and at the intersections of multiple means of marginalization (e.g., gender, ability, age, sexuality, religion) is vital to advancing health equity in population health.

## Health Equity Implications

In this study of 27 structurally excluded HSPRers, three major themes about DEI efforts observed in HSPR in 2022 were identified. We learned that harm and exclusion within HSPR workplace and educational settings and DEI initiatives fail to address the need for institutional change. In addition, HSPRs are uniquely subjected to shifting political contexts that can undermine equity and inclusion. The current state of HSPR threatens the field’s advancement in diversity but more importantly the sense of belonging, inclusion, and equity experienced by those who are structurally excluded and underrepresented. Given recent events in the political and health policy space, the voice of HSPR is needed, especially from those who are representative of the communities who are harmed the most by recent efforts to roll back rights and opportunities (e.g., abortion care, affirmative action, and DEI bans). Without change, we risk losing these voices and further perpetuating structural racism within the field as well as health policy making and practice.

## Abbreviations Used

CEO: chief executive officer
COVID: coronavirus disease
DEI: diversity, equity, and inclusion
HBCUs: Historically Black Colleges and Universities
HR: human resources
HSPR: health services and policy research
LGBTQI+: lesbian, gay, bisexual, transgender, queer and/or questioning, intersex
MD: Medical Doctor
PhD: Doctor of Philosophy
TA: teaching assistant
US: United States
