# Supplementary material for: Navigating Hostile Workplaces and Educational Spaces Within Health Services and Policy Research
Source: Health Equity. 2024 Dec 16;8(1):806–15. doi: 10.1089/heq.2024.0121 (PMC11671308; doi:10.1089/heq.2024.0121)
Supplement: Supplementary Data S1 [file heq.2024.0121_supp_datas1.docx]

**Supplementary Material: Item 1**

| Definitions of Characteristics of White Supremacy Culture | |
| --- | --- |
| Characteristic | Definition |
| Fear | The primary tool used to manipulate individuals. It manifests as a fear of inadequacy, not belonging, and a false sense of safety. White supremacy employs fear to create disconnection among people, both across and within racial identities, fostering divisions for the benefit of a few in terms of power and profit. |
| Either/or and Binary Thinking | is a cognitive approach that frames options or issues as mutually exclusive, such as good/bad, right/wrong, with us/against us. It lacks consideration for both/and possibilities, oversimplifies complex matters, and hinders learning from mistakes or handling conflict. This binary thinking leads to urgency in decision-making, often driven by agendas and goals, discouraging alternative considerations. |
| Worship of the Written Word | is a perspective that places excessive value on written communication and documentation. It disregards alternative forms of knowledge such as stories, embodied knowing, and intuition. Rooted in systemic racism, it prioritizes the writing and wisdom of the white group, adheres to academic standards emphasizing "original" work, and sometimes involves claiming ownership of knowledge for ego satisfaction rather than fostering communal growth. |
| Objectivity | The belief in being neutral and devoid of emotions in decision-making. It enforces linear and logical thought, dismissing alternative perspectives. Objectivity tends to overlook how logical thinking can mask personal emotions and agendas, especially those driven by a fear of losing power or comfort. Additionally, it is used to protect existing power dynamics and maintain the status quo. |
| Individualism | manifests as a focus on oneself as an individual, often neglecting acknowledgment of the influence of dominant identities and cultural norms. There is a desire for individual recognition, valuing competition over cooperation, and limited accountability to peers. Overall, it fosters a culture of isolation, competition, and a failure to appreciate collaborative efforts. |
| Quantity over quality | Prioritizes quantifiable goals, valuing measurable outcomes like attendance, circulation, and finances over the quality of relationships, democratic decision-making, and emotional presence. This mindset often leads to short-term, urgent, and either/or thinking, driven by a consuming effort to achieve numerical goals, disregarding the long-term impact on relationships and the nuanced aspects of decision-making. |
| Power Hoarding | The tendency to withhold and resist sharing power within an organization. It involves viewing power as limited and feeling threatened by suggestions for change, often taking such suggestions personally. |
| Fear of Open Conflict | is the reluctance to engage in expressed conflict, particularly by those in power. It involves avoiding or deflecting issues that cause discomfort, often blaming the person raising the problem rather than addressing the issue itself. |
| A Sense of Urgency | A constant pressure to act quickly, making it challenging to prioritize inclusivity, democratic decision-making, long-term thinking, and consideration of consequences. This urgency often sacrifices potential allies, reinforcing existing power hierarchies and leading to expedient decision-making. |
| Defensiveness | An organization is characterized by a focus on preventing abuse and protecting power rather than fostering individual capacities. This mindset, rooted in either/or and binary thinking, perceives criticism as a threat, making it challenging to introduce new or challenging ideas. |
| Denial | The act of rejecting or disregarding discussions about white supremacy or racism, particularly when raised by racially minoritized individuals. Denial also includes minimizing or ignoring the historical and structural aspects of racism, refusing to acknowledge the emotional toll of racism, and denying racial conditioning. |
| Paternalism | A system where those in power control decision-making, define standards, and assert the existence of one right way. Those with power assume entitlement to make decisions on behalf of those without power, often disregarding the perspectives of the latter. |
| Progress is Bigger | Is a mindset that defines success by continuous growth, often without regard for the organization's mission or the well-being of those it serves. It prioritizes expansion in staff, projects, and influence, emphasizing quantity over quality. The focus is on numerical measures like finances and power, ignoring the value of relational processes and the broader costs in social, emotional, psychic, embodied, spiritual, and financial aspects. |
| Belief in one right way | The belief that there is only one correct method or approach to doing things. It involves the assumption that once introduced to this right way, individuals or groups should adopt it, and failure to do so is seen as a flaw in those who resist conformity. This mindset is comparable to a missionary perspective, where the focus is on imposing one's beliefs rather than recognizing the value in diverse perspectives or ways of living. |
| Right to Comfort | Is the belief that those in power are entitled to emotional and psychological comfort, often prioritizing logic over emotion. This mindset involves scapegoating individuals who cause discomfort by naming issues like racism, rather than addressing the actual problems. |
| Perfectionism | Characterized by a lack of appreciation for others' work, a tendency to focus on inadequacies and mistakes, and an internalized harsh inner critic. It involves the belief in one right way, leading to a failure to recognize and learn from mistakes, and a general difficulty in acknowledging and appreciating what is right or well-done. |
| Qualified | Refers to an internalized characteristic prevalent among educated, middle, and owning class white individuals. It involves the belief that they are duty-bound to fix, save, and set straight the world, often rooted in mainstream Christian ideology. This characteristic can be overt or deviously internalized, leading to an assumption of inherent qualifications to improve situations without recognizing their role in perpetuating issues. |
