# Supplementary material for: Navigating Hostile Workplaces and Educational Spaces Within Health Services and Policy Research
Source: Health Equity. 2024 Dec 16;8(1):806–15. doi: 10.1089/heq.2024.0121 (PMC11671308; doi:10.1089/heq.2024.0121)
Supplement: Supplementary Table S1 [file heq.2024.0121_supp_datas2.docx]

**Supplementary Material Item 2. Health services and policy research participants, by title and type of employment**

|  | | |
| --- | --- | --- |
| **Pseudonym** | **Job Title** | **Employer Type** |
| Jesse | Assistant Professor | Academic Institution |
| Memphis | Associate Professor | Academic Institution |
| Bailey | Program/Site Director | Academic Institution |
| Spencer | Consultant | Did not disclose |
| Ellis | Doctoral Candidate | Academic Institution |
| Jackie | Industry | Healthcare Administration |
| Sevyn | Industry | Healthcare Administration |
| Jamie | Instructor/Director | Academic Institution |
| Mason | Instructor/Director | Healthcare Administration |
| Remy | Industry/Counsel | Non-Academic Research Organization |
| Kaiden | Doctoral Candidate | Academic Institution |
| Aaron | Program Director | Non-Profit |
| Addison | Doctoral Candidate | Academic Institution |
| Amari | Associate Professor | Academic Institution |
| Wren | Organizer/Consulting | Academic Institution |
| Adrian | Professor | Academic Institution |
| Parker | Postdoctoral Fellow | Academic Institution |
| Erin | Government | Government |
| Arden | Assistant Professor | Academic Institution |
| Ashton | Associate Professor | Academic Institution |
| Drew | Government | Government |
| Sage | Assistant Professor | Academic Institution |
| Laken | Director | Non-Academic Research Organization |
| Yael | Professor/Director | Healthcare Administration |
| Cameron | Postdoctoral Fellow | Academic Institution |
| Paxton | Consultant | Did not disclose |
| Ryan | President and Chief Executive Officer | Non-Academic Research Organization |
